# Can We Have Guidelines or Just Guidance for Rare Fungal Infections?

**DOI:** 10.3390/jof11090666

**Published:** 2025-09-11

**Authors:** Nancy N. Vuong, Dimitrios P. Kontoyiannis

**Affiliations:** 1 Division of Pharmacy, The University of Texas MD Anderson Cancer Center, Houston, TX 77030, USA; nnvuong@mdanderson.org; 2 Division of Internal Medicine, Department of Infectious Diseases, Infection Control and Employee Health, The University of Texas MD Anderson Cancer Center, Houston, TX 77030, USA

**Keywords:** rare or uncommon invasive fungal infections, guidelines, guidance

## Abstract

In this perspective, we discuss the limitations of medical guidelines as it relates to the management of uncommon invasive fungal infections (IFIs) or infrequent manifestations of more common IFIs. We emphasize the difficulties to define “gold standards” for diagnostics and treatment based on limited and low-quality evidence. We posit that such “guidelines” based on scarce data may be suboptimal and could be in some cases even harmful. Specifically, guidelines are often seen as rigid rules to follow which can prevent a critical examination of the nuanced management of individual patients with rare IFIs. We also emphasize that guidelines are often not updated frequently enough and therefore may not reflect the current treatment landscape. For all those reasons, we suggest that the term “guidance” may be more appropriate than “guidelines” for rare IFIs. Finally, we pose several questions regarding constructing future “Guidelines”/“Guidance for such entities”.

What constitutes a guideline is controversial and has spurred debates in their relevance to medical practices [1,2,3]. Although written with good intentions and providing notable benefits, they could also pose distinct challenges to heath care providers, patients, and heath care utilization [4]. Specifically, guidelines could inadvertently steer prescribing practices down potentially uncertain paths and often perpetuate the mentality of groupthink and agreeable narratives anchored on a less-than-compelling evidence base. One has witnessed those conundrums with the surviving sepsis campaign, management of cardiovascular diseases, and even dosing schemes of antimicrobial guidelines [5,6,7]. The practice of indiscriminately conforming to published guidelines has led to “mandates,” influenced billing practices, and set tones for “standards of care.” The debate intensifies when guidelines are developed for uncommon diseases. In these unfamiliar scenarios, providers turn to the best available information for guidance of guidelines, which are written based on limited literature of anecdotal cases; small, retrospective, often-single-institution studies; or registries that might be unsuitable for guideline construction [8,9,10,11]. However, the best use for uncontrolled small series is the description of toxicities of therapies, recognition of epidemics, and initial identification of unrecognized syndromes [12].

Several recently published manuscripts offer global guidelines for the diagnosis and management of rare opportunistic yeast and mold infections [13,14,15,16,17,18] (Figure 1), which has compounded widespread “guideline fatigue.” The authors of these guidelines are to be commended for summarizing a large volume of literature and for trying to offer their best practical guidance for very complex and unusual mycoses. Admittedly, the senior author of this editorial has participated as a coauthor or senior author of “guidelines” focusing on rare mycoses, or from commenting on the treatment of unusual manifestations or clinical scenarios of common mycoses [15,16,17,18].

Many issues exist for guidelines for rare mycoses, which are multifactorial. Specifically, case reports, short case series, and synthesis of data from registries are challenged by publication and reporting biases. For example, there is no denominator nor is there a sense of what is reported as the typical manifestations of a particular rare fungal disease [19,20,21]. The heterogeneity and complexity of such entities is significant [22]. The same argument can be made for uncommon manifestations of the most frequent mycoses such as candidiasis, aspergillosis, and cryptococcosis where the pivotal randomized controlled studies focused on the predominant clinical manifestations of those mycoses (i.e., fungemia for Candida, lung infections for *Aspergillus*, and meningitis in *Cryptococcus* [23,24,25,26,27,28]). Specifically, there is a paucity of quality data to inform the practice of unusual clinical manifestations of common mycoses (e.g., *Candida endocarditis* [29]), and those are subject to the same uncertainties seen with developing therapeutic recommendations of rare mycosis.

**Figure 1:**
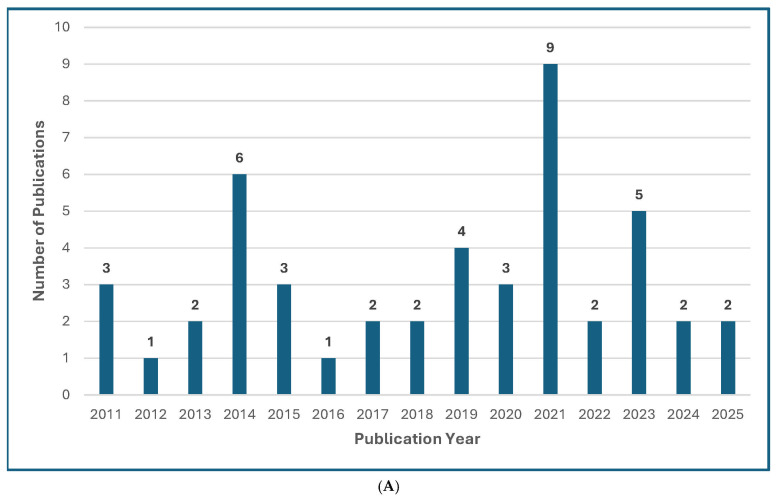

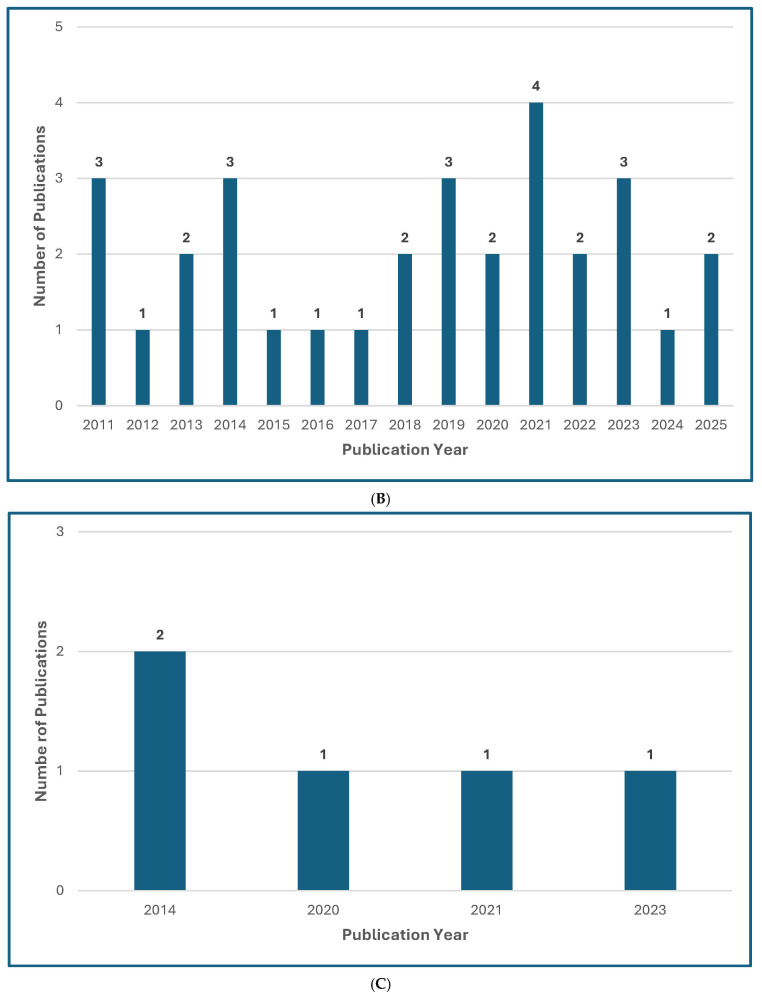

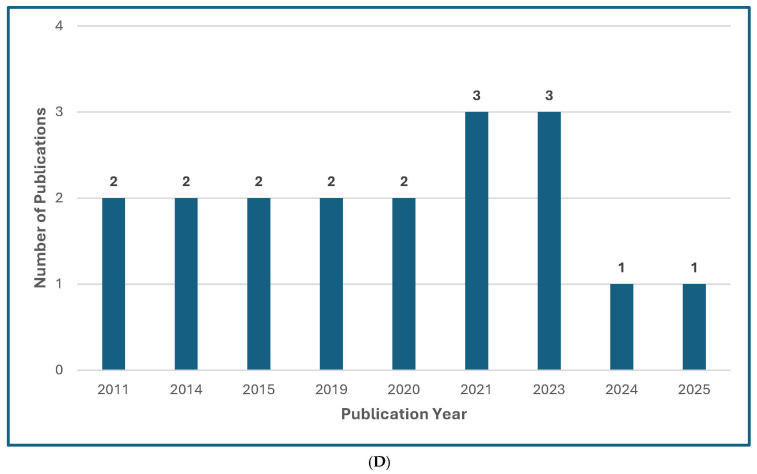
Treatment Guideline Trends for Unusual or Rare Fungi. (**A**) Trend of Publications on Treatment Guidelines for Unusual Fungal Infections, *n* = 47 [13,14,18,30,31,32,33,34,35,36,37,38,39,40,41,42,43,44,45,46,47,48,49,50,51,52,53,54,55,56,57,58,59,60,61,62,63,64,65,66,67,68,69,70,71,72,73]. (**B**) Trend of Publications on Treatment Guidelines Mentioning Mucormycosis, *n* = 31 [18,31,33,34,35,36,39,40,41,42,43,44,48,50,51,52,53,54,55,56,57,61,63,64,65,66,67,68,69,71,73]. (**C**) Trend of Publications on Treatment Guidelines Mentioning Only Rare Molds (not Aspergillus, not Mucorales), *n* = 5 [14,38,60,62,72]. (**D**) Trend of Publications on Treatment Guidelines Mentioning Rare Yeasts, *n* = 18 [13,30,31,33,37,38,41,42,45,47,55,58,59,60,62,65,67,70]. A qualified medical librarian conducted a comprehensive search of literature published from 2005 to 2025. Medline (Ovid), Embase (Ovid), and Scopus were queried using both natural language and controlled vocabulary terms for clinical guidelines, recommendations, consensus statements, and best practices; relevant mold and yeast organism terms; and terms related to international, national, and professional medical societies. After deduplication, 77 relevant results were identified. We excluded guidelines not available in English and pediatric guidelines and included a total of 47 guideline publications.

To compound the challenges, it is unclear at times if those unusual fungi reported in case reports, case series, or registries contain misidentified fungi. Those misclassification errors could result in erroneous conclusions. The difficulties in proper identification and differentiation of some of these uncommon fungi on morphological grounds is at times challenging for the average clinical laboratory, as these labs do not have the critical volume of cases and the expertise in identification of uncommon fungi. Although rapid diagnostic platforms, such as Matrix-Assisted Laser Desorption/Ionization Time-of-Flight mass spectrometry (MALDI-TOF), holds promise as a fast and accurate identification tool for fungal identification, its performance might be less robust for the identification of uncommon fungi due to lack of expanded libraries for such fungi [74]. The problem of fungal identification is further amplified in low- and middle-income countries (LMICs) that often lack the appropriate infrastructure for reliable identification of fungi, especially the uncommon fungi [75]. Of note, such global guidelines have been developed to promote diagnosis and management of invasive fungal infections (IFIs), including rare IFIs in LMIC [76]. Unless these fungi are reliably identified by molecular phylogenetic methods, classification biases remain a concern.

In addition to all these quandaries, unusual fungal infections afflict a very heterogeneous population at risk, ranging from immunocompetent patients (e.g., mold infections such as mucormycosis following trauma, rare yeast infections in intravenous drug users) to patients with profound and complex defects in a net state of immunosuppression. Most of these infections have no surrogate biomarkers for early diagnosis. Additionally, there is a diversity of affected sites, and the correlation of cell-free in vitro susceptibility with outcomes is difficult to ascertain [77]. Additional confounders are the common co-infections [76], especially in high-risk patients [78], and the “survival bias” in patients who recover from neutropenia or have their metabolic effect corrected [22].

Treatment recommendations are even more problematic to be codified into “guidelines” of uncommonly reported and intrinsically resistant yeasts [79] and molds [18]. Many such patients are treated with a variety of combination and salvage therapies, making it increasingly difficult to discern which antifungal therapy should really be used. Importantly, very ill patients with uncommon opportunistic mycoses have multiple interventions occurring simultaneously or sequentially [22], including surgery, and do not account for the activity of agents in the context of continuous immunosuppression [80]. This adds several levels of complexity in assessment. For example, fusariosis seen at the University of Texas MD Anderson Cancer Center has been an uncommon infection typically seen in patients with relapsed or refractory hematologic cancers, and its outcome is predominantly host-driven [81]. The lack of association between antifungal in vitro susceptibilities with fusariosis outcomes demonstrates the intricacies of interpreting laboratory data and fusariosis management [22,77,82], where poor host immunity was the key decider of antifungal response, especially for opportunistic mycoses.

Finally, guidelines are not routinely published on a reliable schedule. Oftentimes, it takes several years, sometimes nearly a decade, for guidelines to update. For example, the ESCMID and ECMM guidelines for rare yeasts were published in 2014 and were not updated until 2021 [13,30]. The delayed process of reviewing and publishing also means that information is behind the times and does not account for the best currently available data. This frequently leaves out potential advances in therapy of the newer promising agents [83].

We believe that a better term might be guidance instead of guidelines and that providers should have a healthy cautionary approach when interpreting published “guidelines” without the nuances of patient-level individualization [51]. Several interesting questions remain when one constructs a thoughtful guidance/guideline for mycoses in general, and even more importantly, for rare mycoses (Table 1).

## Figures and Tables

**Table 1:** Questions regarding constructing fungal “Guidelines”/“Guidance”.

Who should be in an expert panel? Should we also include, in addition to clinical investigators, practicing clinicians who have a “critical mass” of expertise?
Should we strive for more diverse panels that include experts from different specialties (e.g., hematologists) and frontline primary care physicians?
How do we mitigate conflict of interests in guideline panels, in view of the recent introduction of several new drugs with activity against rare fungi?
Can we develop quality scores to measure compliance to guidelines for rare yeasts and molds [84,85,86]? If so, how we weigh and aggregate factors to measure clinical impact based on guideline adherence?
Would there be a role for AI-generated treatment guidelines rare yeasts and molds [87]? If so, how guidelines generated by large language models, aim to provide personalized recommendations for uncommon and complex clinical scenarios?
Should there be a core reporting dataset as a minimum standard in the literature and registry reports to use data of infections by rare fungi as data to construct guidelines [88]?
Should guidelines take more into questions heath care utilization and operational efficiencies?

## Data Availability

No new data were created or analyzed in this study. Data sharing is not applicable to this article.
